# Visual and electrical degradation data of five years aged rooftop photovoltaic modules

**DOI:** 10.1016/j.dib.2020.105762

**Published:** 2020-05-24

**Authors:** M Abul Hossion

**Affiliations:** Department of Physics, BSMR Maritime University, Dhaka 1216, Bangladesh

**Keywords:** Ageing, degradation, photovoltaic modules, silicon, efficiency, solar cell

## Abstract

Sun light as a renewable energy resource is getting popular day by day. Power production from a solar power plant is extensively dependent on the weather condition, daylight duration, available sunlight, air quality etc. Thus installation of large scale solar power plant requires long term feasibility study, previous five years weather data, lifetime of the solar panel, projected maintenance requirement and man power. In this survey we have visited several sites of roof top solar power plants in the Dhaka city which are aged at least five years. Firstly, we have conducted visual study of the solar panels on the roof top for visible degradation due to environment and ageing. Then we have measured Current-Voltage characteristics under sun light using portable PV-200 Seaward I-V Tracer. The Current-Voltage data were analyzed using ‘Seaward Solar Chart’ data analysis tool. The tool was used to plot Current-Voltage and Power-Voltage curves. From the data we have estimated the Standard Test Condition (STC) power, Fill Factor (F.F) and Efficiency of selected photovoltaic modules. To get a clear view over the experience of installed rooftop solar photovoltaic modules in Dhaka city, the data will be useful. The data will help us to project the challenges and provide a guide line to maintain an economically viable solar photovoltaic installation.

Specifications table

**Table:** 

Subject	Renewable Energy, Sustainability and the Environment
Specific subject area	Roof top silicon photovoltaic module's visual and electrical degradation due to ageing (five years).
Type of data	Table, Image, Figure.
How data were acquired	Rooftop PV modules survey Instruments: i) Solar I-V Tracer, ii) Solar irradiation flux meter Model and make of the instruments used: i) PV 200, ii) Solar Survey 200R Seaward Electronic Ltd, UK. Software: SolarCert software tool for data transfer and data processing from Seaward Electronic Ltd, UK. Instruments: DSLR Camera Model and make of the instruments used: EOS 700D, Canon.
Data format	Raw data
Parameters for data collection	i) Visual inspection, ii) current-voltage measurement under nearly one sun irradiation, iii) Solar irradiation flux iv) PV module tempeprature
Description of data collection	These data were collected by visiting the various roof top of buildings of Dhaka city.
Data source location	City: Dhaka, Country: Bangladesh, Location: Mirpur-12.
Data accessibility	With the article

## Value of the data

• The survey data of rooftop PV modules in the city of Dhaka provides base line information about the modules for future surveys.
• The survey provides data on PV installations on the rooftop of residential and commercial buildings which can be followed for contrast in maintenance practices.
  The survey covers one installation of Mono-Silicon PV modules, two installations of Poly-Silicon PV modules and one installation of thin film Silicon PV modules, thus will provide comparison between performances of different technologies in the local environments.
• The survey is based on visual observations and electrical measurements.
• PV Modules in the study are relatively young (0 to 5 years) which can be followed over the years to trend the degradation in roof top PV installations with aging in hot and humid environments of Dhaka.

## 1 Data Description

**Fig. 1:**
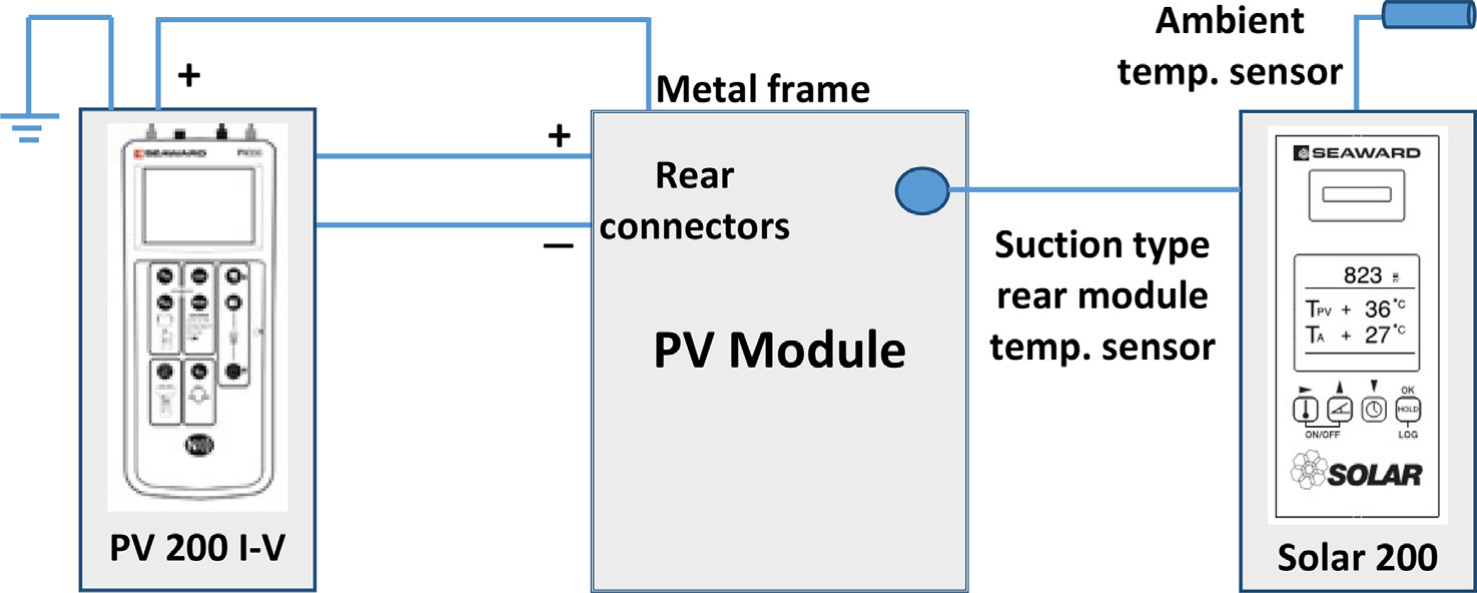
Electrical connections showing the measurement of current-voltage, isolation resistance, solar irradiance, and temperature of PV module during survey.

**Fig. 2:**
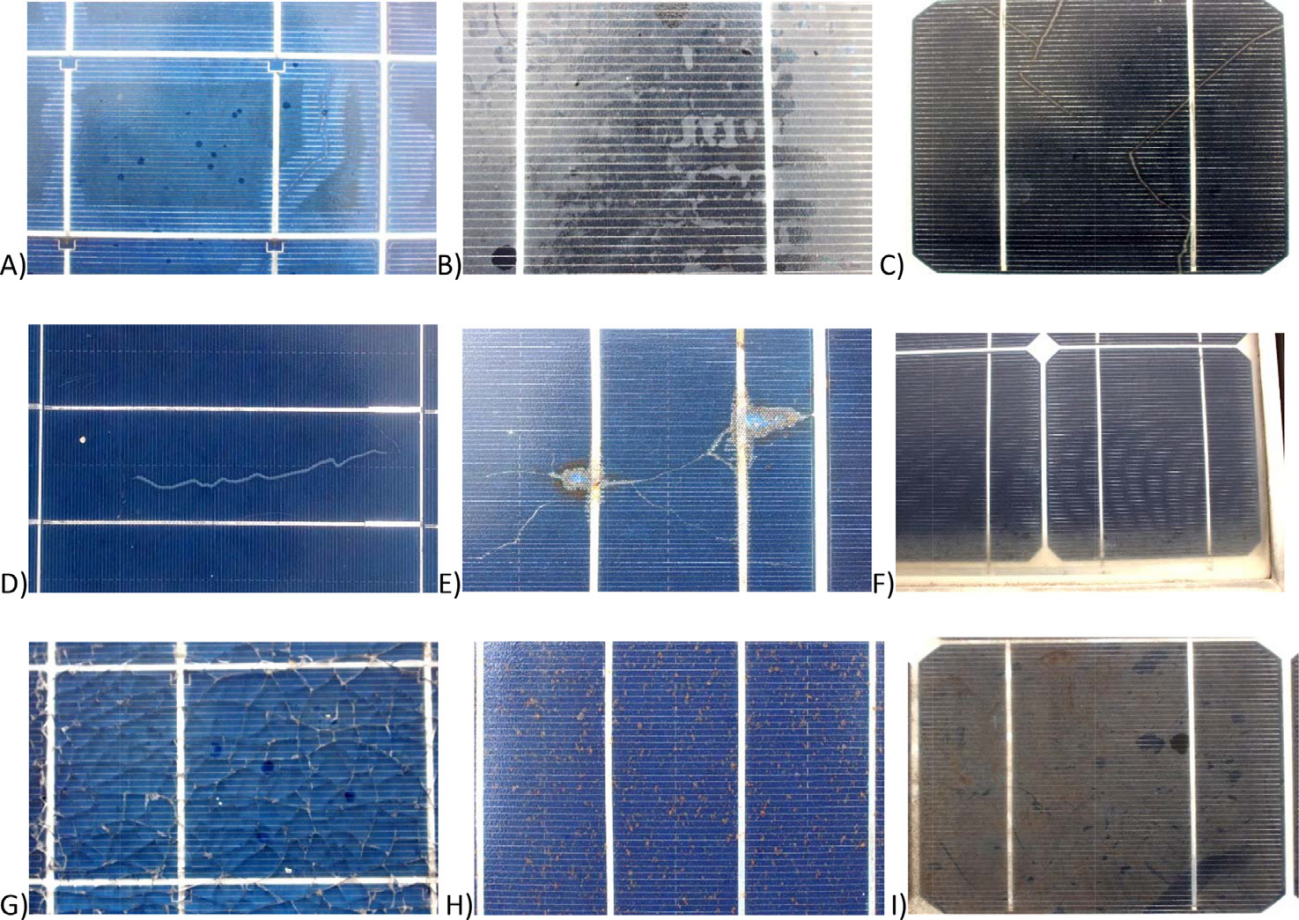
A) PV02_Poly_50W, Discoloration of metal contacts from shiny silver to brown, B) PV05 _Mono_100W, Delamination of cell from polycrystalline blue to brown, C) PV05_Mono _100W, D) PV07_Poly_150W, Visible snail trails, E) PV06_Poly_150W, Visible cracks, F) PV05_Mono_ 100W, Fogged glass at the edge of panel, G) PV02_Poly_50W, Shattered glass, H) PV06_Poly_ 150W, Brown spots on front glass, I) PV05_Mono_100W, Dust accumulated on the front glass.

**Fig. 3:**
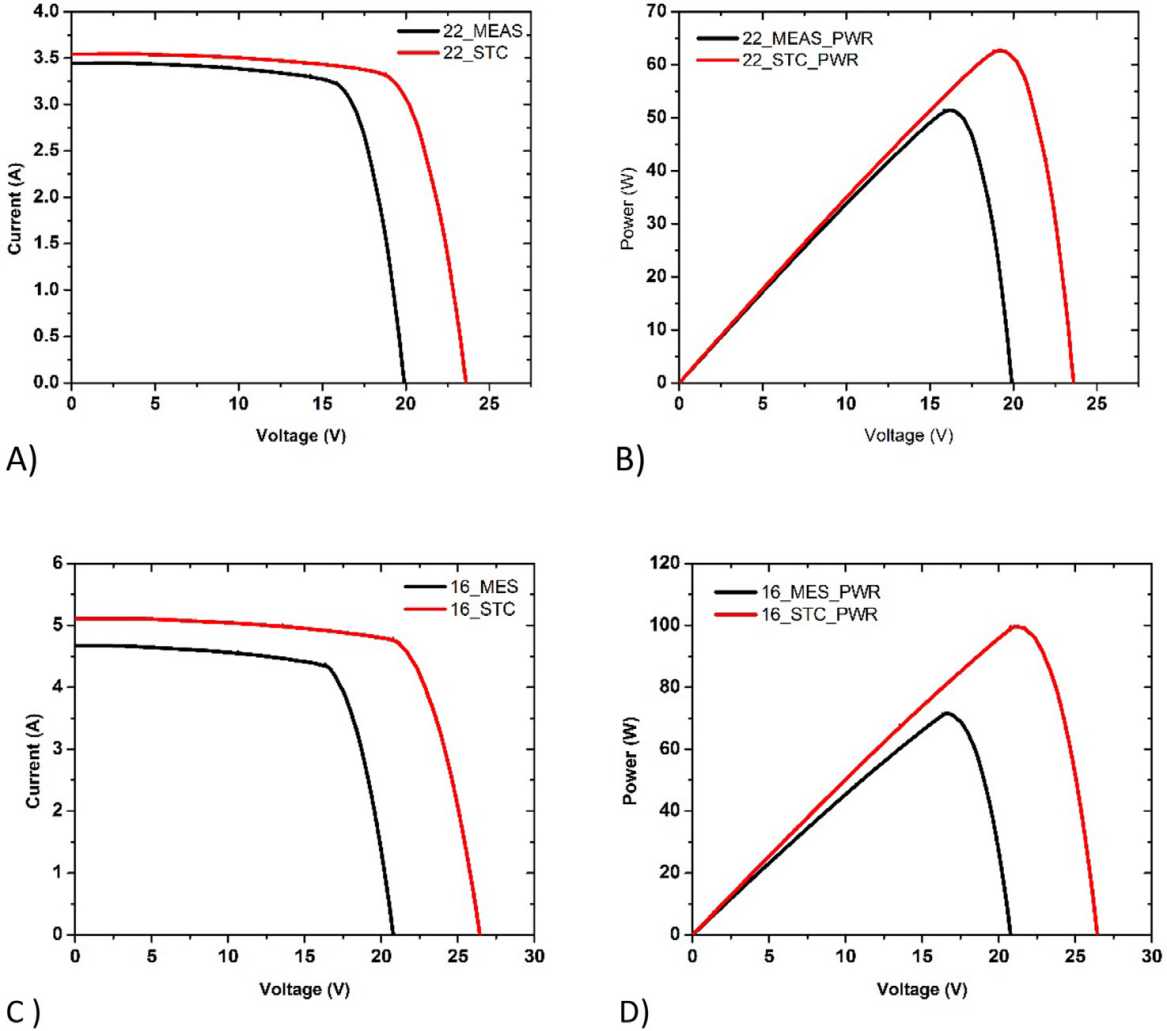
Current-voltage (I-V) and Power-voltage (P-V) data of newly purchased PV modules (A&B) 22_PV03_Poly_100W, under solar irradiance 944Wm^−2^, (C&D) 16_PV01_Mono_100W, under solar irradiance 898Wm^−2^ respectively.

**Fig. 4:**
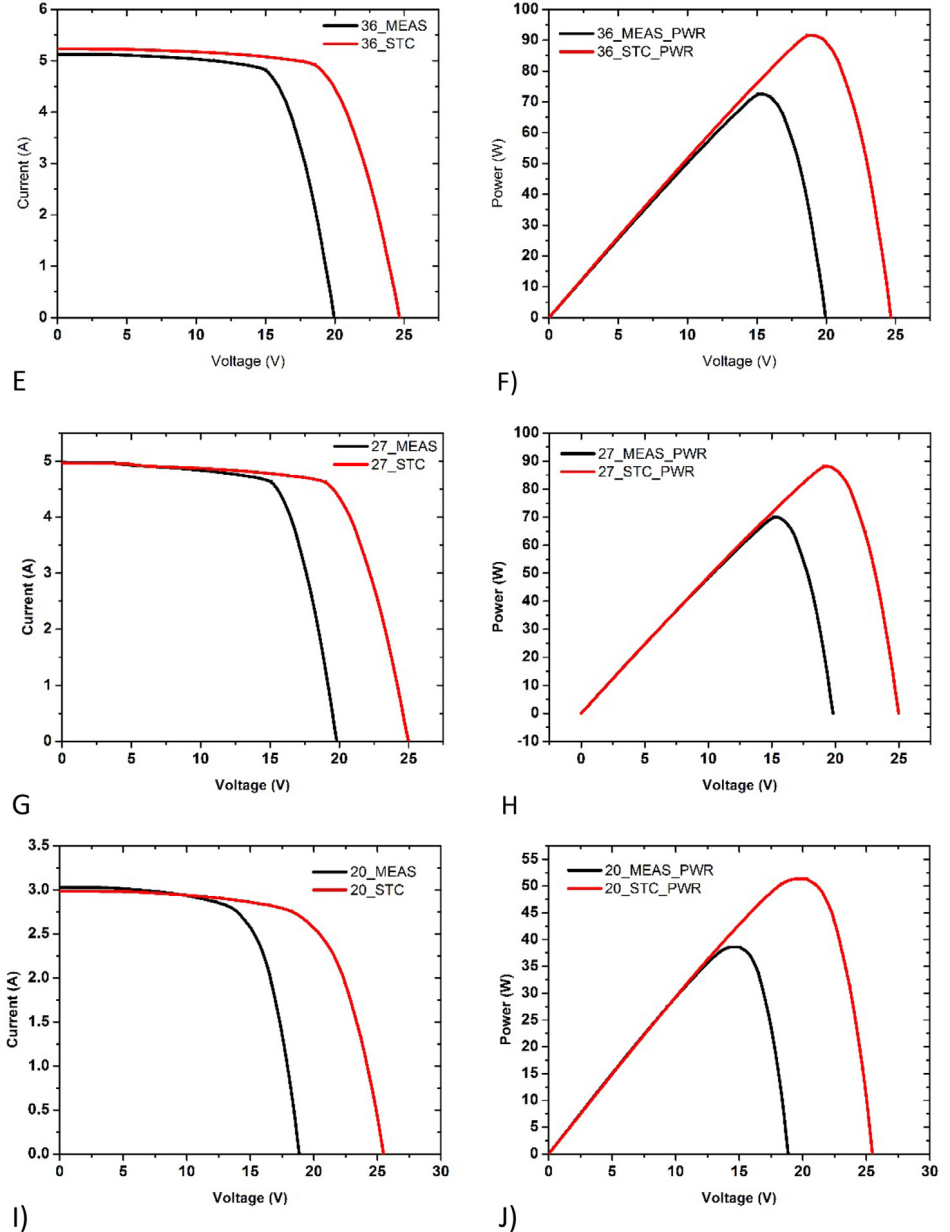
Current-voltage (I-V) and Power-voltage (P-V) data of E&F) 36_PV05_MonoSi_100W Cleaned, under irradiance of 959Wm^−2^, G&H) 27_PV05_MonoSi_100W_ Dust covered, under irradiance of 980Wm^−2^, I&J) 20_PV02_PolySi_50W Cleaned, under irradiance of 962Wm^−2^ respectively.

**Fig. 5:**
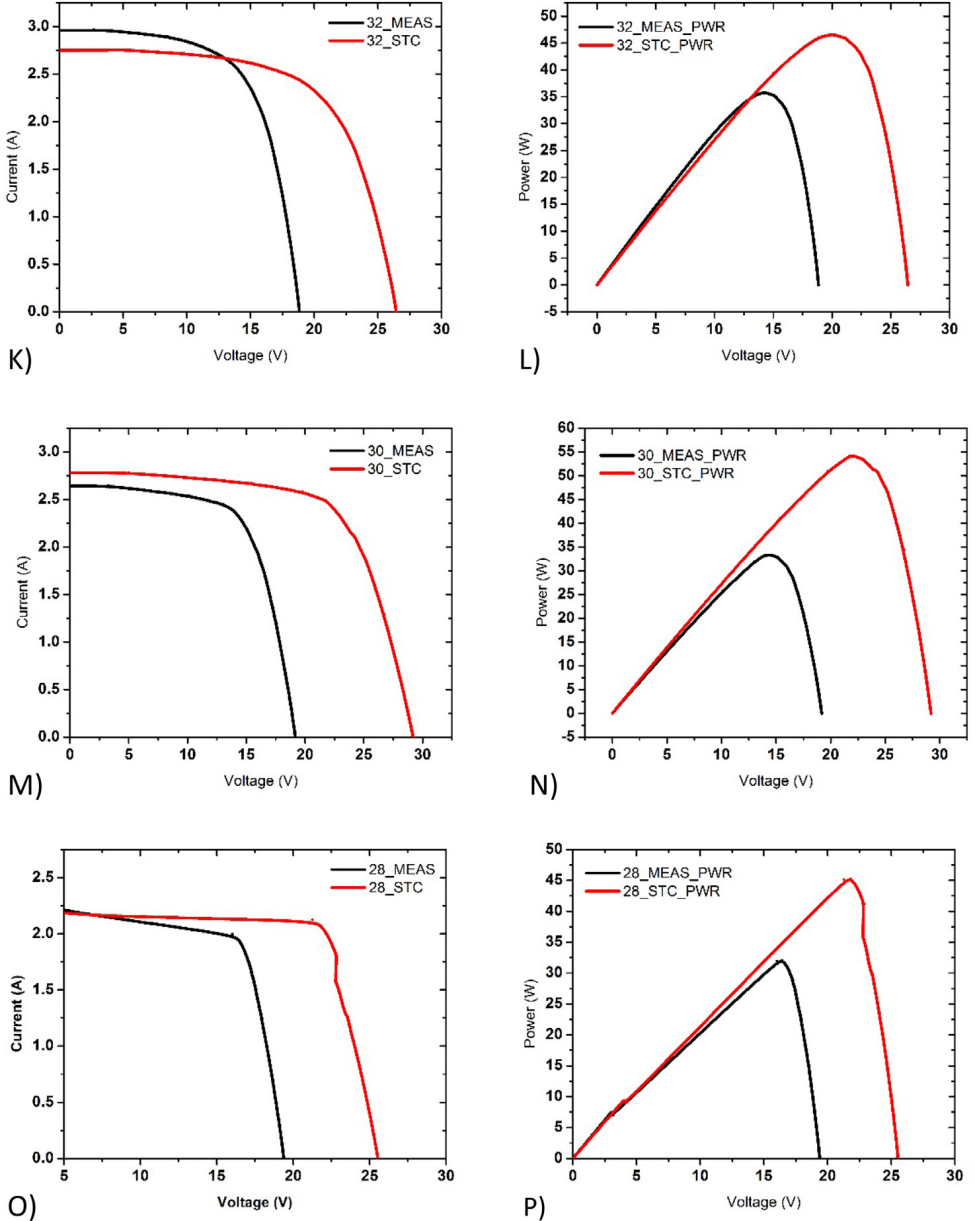
Current-voltage (I-V) and Power-voltage (P-V) data of (K&L) 32_PV02_PolySi_50W Dust covered, under irradiance of 1028Wm^−2^, (M&N) 30_PV02_PolySi_50W Snail Trails, under irradiance of 907Wm^−2^, (O&P) 28_PV02_PolySi_50W Shattered glass, under irradiance of 1019Wm^−2^ respectively.

**Fig. 6:**
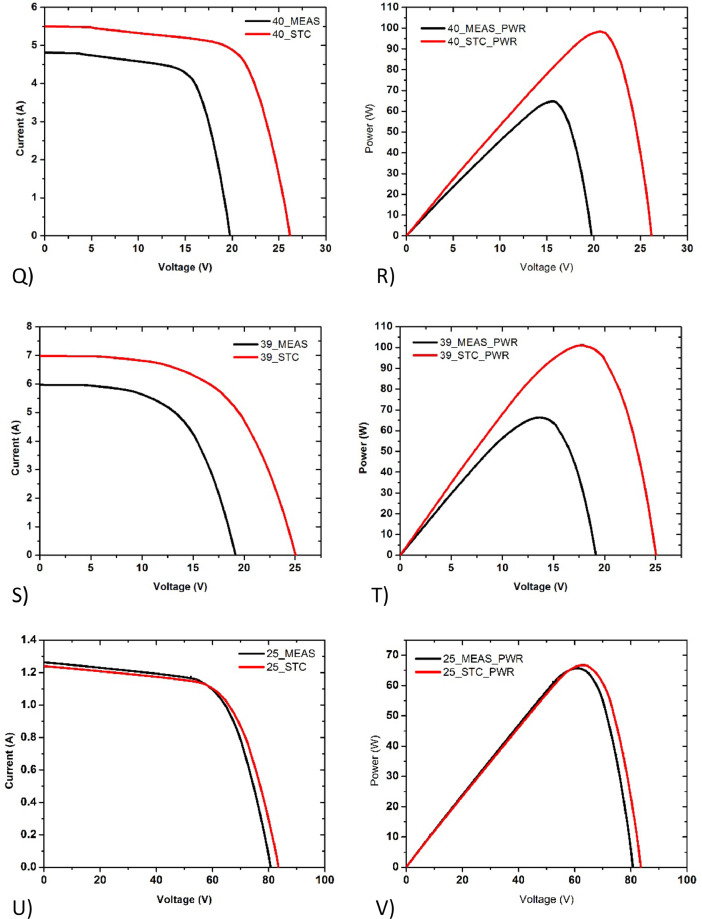
Current-voltage (I-V) and Power-voltage (P-V) data of (Q&R) 40_PV06_PolySi_150W Clean front glass, under irradiance of 849Wm^−2^, (S&T) 39_PV06_PolySi_150W Snail trails, under irradiance of 834Wm^−2^, (U&V) 25_PV04_Thin film_100W, under irradiance of 912Wm^−2^ respectively.

The data set in this article describes the visual degradation and electrical performance of photovoltaic (PV) modules installed on the rooftop of buildings in Dhaka city. Table 1 describes the information on the PV modules which were inspected during the survey. Fig. 1 shows the electrical connections used in the measurement of current-voltage and isolation resistance of PV module during the survey. Fig. 2 provides the visual degradation images of PV modules surveyed. Table 2 describes the electrical data, solar irradiation and temperature of PV modules surveyed. Fig. 3-6 shows the current-voltage (I-V) and power-voltage (P-V) plots of PV modules surveyed. The numerical data and information of the PV installations are given as suplimentary document with the article.

**Table 1:** Information on PV modules surveyed during the survey.

Serial no	PV modules code	Type of silicon solar panel	Rated Power, W	Manufacturing year and Age	Physical Condition
1	16_PV01	Mono crystalline	100	2019/New	Clean, Transparent
2	22_PV03	Polycrystalline	100	2019/New	Clean, Transparent
3	25_PV04	Silicon thin film	100	2015/4	Clean, Weak metal frame
4	20_PV02	Polycrystalline	50	2014/5	Clean, Transparent
5	28_PV02	Polycrystalline	50	2014/5	Shattered front glass
6	30_PV02	Polycrystalline	50	2014/5	Snail Trail, Discolor EVA
7	32_PV02	Polycrystalline	50	2014/5	Dust and oil front glass
8	27_PV05	Mono crystalline	85	2014/5	Dust and oil front glass
9	36_PV05	Mono crystalline	85	2014/5	Cleaned front glass
10	39_PV06	Polycrystalline	150	2013/5	Snail Trail
11	40_PV06	Polycrystalline	150	2013/5	Cleaned front glass

**Table 2:** Electrical data on silicon PV modules surveyed.

Solar Panel age/type	PV Modules Efficiency Table													
	PV modules code	V_oc_ / volt	I_sc_ / Amp	Irradiance (W/m^2^)	Temperature of PV modules°C	Fill Factor %	V_mpp_ (V)	I_mpp_(A)	Cell Area/ m^2^	Rated Power/W	STC Power / W	Measureed Power/W	Efficiency %	Remark
New/ Mono	16PV01	20.8	4.675	898	54	73.75	16.64	4.3	0.52	100	98.77	71.71	15.36	Clean
New/Poly	22PV03	19.9	3.444	944.6	45	75.01	16.23	3.2	0.63	100	62.70	51.41	8.64	Clean
5y/Poly	20PV02	18.9	3.029	961.8	57	67.71	14.42	2.7	0.35	50	51.35	38.76	11.51	Cleaned
5y/Poly	28PV02	19.4	2.485	1019	56	66.52	16.44	1.9	0.35	50	45.23	32.07	8.99	Shattered Glass
5y/Poly	30PV02	19.2	2.642	906.8	68	65.71	14.57	2.3	0.35	50	54.12	33.33	10.50	Snail Trail
5y/Poly	32PV02	18.8	2.958	1028	61	64.16	14.25	2.5	0.35	50	46.55	35.68	9.92	Not Cleaned
5y/Mono	27PV05	19.8	4.98	979.8	52	71.1	15.1	4.6	0.56	85	88.33	70.11	12.78	Dust
5y/Mono	36PV05	20	5.127	959.2	50	71.03	15.05	4.8	0.56	85	90.94	72.83	13.56	Cleaned
5y/Poly	39PV06	19.2	5.969	834.1	54	58.05	13.72	4.8	0.71	150	100.86	66.53	11.23	Snail Trail
5y/Poly	40PV06	19.8	4.815	849.1	56	68.17	15.65	4.1	0.71	150	98.53	64.99	10.78	Cleaned
5y/Thin film	25PV04	80.7	1.263	911.8	42	64.6	60.72	1.1	0.9	100	66.86	65.84	8.02	Cleaned

## 2 Experimental Design, Materials, and Methods

The survey on solar PV module was conducted in field especially on the rooftops of the residential and commercial buildings in Dhaka city. We have surveyed the PV installations which are aged five years to be able to observe the degradation of PV modules [1].

A solar PV characterization tool kit was taken to the field for detailed inspection of the modules. The characterization tool kit contains cleaning materials, current-voltage (I-V) measurement equipment, solar irradiation flux meter with panel and ambient temperature sensor and digital camera. The primary inspection also includes measurement of cell area, cell numbers, manufacturer detail, standard testing condition data by the manufacturer, installation date etc.

After conduction of visual inspection we have isolated the solar system from the grid. Then few panels were isolated from the array to be investigated depending on the visual inspection and ageing features. We have used the PV 200 meter to measure the i) current-voltage and ii) isolation resistance data under nearly one sun using the following circuit shown in Fig. 1. The Solar Survey 200R solar irradiation flux meter was remotely connected to the PV 200 meter which measures the i) solar irradiation, ii) ambient temperature and iii) the temperature of the rear part of PV module.

We have ensured that all the data was taken under the solar irradiation was 850W/m^2^ and above. Following this procedure we have taken 5 sets of data for each panel. The variation of the solar irradiation was kept 10% or less which was mostly due to cloud shading. All the data was taken during the day time around 12.00 pm to 14.00 pm. The I-V data were taken before and after cleaning the panel to observe the current loss due to dust accumulation.

The data processing was performed using Seaward Solar Chart tool. The transfer protocol was completed by using SolarCert data logger software tool. After transferring the raw data file in the computer, the processing of the data is performed by SolarCert software. The software allows us to plot current-voltage (I-V) graph and power-voltage (P-V) graph under nearly one sun and under Standard Testing Condition (STC).

### 2.1 PV Modules Survey Data

Table 1.

### 2.2 Visual Degradation Data

Visual inspection is a primary way of identifying degradation of a solar cell. This provides a very quick analysis of the panel health condition at a glance. Most of the visual degradation can be tailed back to further deterioration of the electrical and physical damage [2,^3^,4]. During the survey we have observed various degradations such as discoloration, front side delamination, snails track, cracks, glass degradation, dust accumulation which are shown in Fig. 2.

### 2.3 Electrical Degradation Data

The electrical analysis of photovoltaic modules allows us to estimate the power generation and average degradation of the PV modules per annual. The I-V measurement was used to calculate Fill Factor (F.F) and Efficiency [5] using the following equations

$$\text{Fill}\;\text{Factor}\;=\;\frac{I_{m}V_{m}}{I_{sc}V_{oc}},\;\text{Efficiency}\;=\;\frac{F.F\;I_{sc}V_{oc}}{P_{in}}=\frac{F.F\;I_{sc}V_{oc}}{irradiance\;\times \;cell\;area}$$

Here, I_m_ is current and V_m_ is voltage at maximum power point, I_sc_ is short circuit current, V_oc_ is open circuit voltage, P_in_ is input power received by the PV module from the solar radiation, F.F is fill factor, cell area is the sum of total area of all the cells in a PV module and irradiance is the amount of instantaneous solar radiation per unit area during the measurement.

The STC power [6] of the PV Modules were calculated using Seaward Solar Chart software tool.

In this survey we have considered two new PV modules i) 100W Polycrystalline Silicon ii) 100W Mono crystalline silicon as reference panel. The electrical characterization was conducted on various types of panel as available in the field. We have surveyed i) Mono crystalline silicon ii) Polycrystalline silicon and iii) Silicon thin film PV modules.

#### 2.3.1 Reference Silicon PV Module

We have used two new solar PV modules procured from the market as reference. These types of panels are the widely used in the field hence provide us with the initial conditions of the solar PV modules. The I-V and P-V plot are shown in Fig. 3 (A&B) for 22_100W Polycrystalline silicon PV module. The I-V and P-V plot are shown in Fig. 3 (C&D) for 16_100W Mono crystalline silicon PV module.

#### 2.3.2 Silicon PV Module in field

We have surveyed one mono crystalline silicon PV installation. The I-V data and P-V data shown in Fig. 4 (E&F) is for 36_100W Mono Silicon solar PV panel. The panel was cleaned using standard cleaning procedure and dried under sun before the measurement was conducted. The I-V data and P-V data showed in Fig. 4 (G&H) is for 27_100W Mono Silicon solar PV panel before cleaning.

Poly crystalline silicon solar panels are widely used in Bangladesh due to their low cost. Most of our findings in field regarding ageing effect are of polycrystalline solar panels. The I-V and P-V plots of poly crystalline silicon PV modules are given in Fig. 4 (I&J), Fig. 5, Fig. 6 (Q&R) (S&T).

The thin film PV modules are rarely installed in field. We have found particularly one installation which were operational during the survey. The I-V and P-V plots of silicon thin film PV module is shown in Fig. 6 (U&V).

## Declaration of Competing Interest

The authors declare that they have no known competing financial interests or personal relationships which have, or could be perceived to have, influenced the work reported in this article.

## Appendix Supplementary materials

Image, application 1

Image, application 2

Image, application 3

Image, application 4

## References

[1] Jordan D.C., Kurtz S.R. (2013). Photovoltaic Degradation Rates—an Analytical Review. Progress in Photovoltaics: Research and Applications.

[2] Chattopadhyay S., Dubey R., Kuthanazhi V., John J.J., Solanki C.S., Kottantharayil A., Arora B.M., Narasimhan K.L., Kuber V., Vasi J., Kumar A., Sastry O.S. (2014). Visual Degradation in Field-Aged Crystalline Silicon PV Modules in India and Correlation With Electrical Degradation. IEEE Journal of Photovoltaics.

[3] Quintana M.A., King D.L., McMohan T.J., Osterwald C.R. (2002). Commonly observed degradation in field-aged photovoltaic modules. Conference Record of the Twenty-Ninth IEEE Photovoltaic Specialists Conference, New Orleans, LA, USA.

[4] Spataru S.V., Sera D., Hacke P., Kerekes T., Teodorescu R. (2016). Fault identification in crystalline silicon PV modules by complementary analysis of the light and dark current–voltage characteristics. Progress in Photovoltaics: Research and Applications.

[5] Sze S.M., Lee M.K. (2010). Semiconductor Devices: Physics and Technology.

[6] Pérez L., Coello J., Lorenzo E. (2019). Control of the STC power in PV modules’ supplies for utility scale plants. Progress in Photovoltaics: Research and Applications.

