# Supplementary material for: Visual and electrical degradation data of five years aged rooftop photovoltaic modules
Source: Data Brief. 2020 May 24;31:105762. doi: 10.1016/j.dib.2020.105762 (PMC7267713; doi:10.1016/j.dib.2020.105762)
Supplement: Supplementary file 1 [file mmc1.docx]

**I-V & P-V Data**

**Data table for the graph shown in figure 2-5**

**Table 1**

| Voltage | Current | Voltage | Current | Voltage | Power | Voltage | Power |
| --- | --- | --- | --- | --- | --- | --- | --- |
| V | A | V | A | V | W | V | W |
| 16_MEAS | 16_MEAS | 16_STC | 16_STC | 16_MEAS_PWR | 16_MEAS_PWR | 16_STC_PWR | 16_STC_PWR |
| 0 | 4.675 | 0 | 5.11077 | 0 | 0 | 0 | 0 |
| 3.616 | 4.669 | 4.59548 | 5.10421 | 3.616 | 16.8831 | 4.59548 | 23.4563 |
| 3.899 | 4.658 | 4.95604 | 5.10325 | 3.899 | 18.1615 | 4.95604 | 25.2919 |
| 4.814 | 4.645 | 6.1191 | 5.08901 | 4.814 | 22.361 | 6.1191 | 31.1401 |
| 10.652 | 4.57 | 13.5398 | 5.00684 | 10.652 | 48.6796 | 13.5398 | 67.7916 |
| 16.339 | 4.375 | 20.7686 | 4.7932 | 16.339 | 71.4831 | 20.7686 | 99.5479 |
| 16.639 | 4.306 | 21.1499 | 4.7176 | 16.639 | 71.6475 | 21.1499 | 99.7768 |
| 16.856 | 4.23 | 21.4257 | 4.63434 | 16.856 | 71.3009 | 21.4257 | 99.2941 |
| 17.054 | 4.154 | 21.6881 | 4.57776 | 17.054 | 70.8423 | 21.6881 | 99.2828 |
| 17.224 | 4.059 | 21.9043 | 4.47307 | 17.224 | 69.9122 | 21.9043 | 97.9793 |
| 17.376 | 3.994 | 22.0976 | 4.40144 | 17.376 | 69.3997 | 22.0976 | 97.2611 |
| 17.542 | 3.922 | 22.3087 | 4.32209 | 17.542 | 68.7997 | 22.3087 | 96.4202 |
| 17.68 | 3.832 | 22.4842 | 4.22291 | 17.68 | 67.7498 | 22.4842 | 94.9487 |
| 17.788 | 3.756 | 22.6215 | 4.13916 | 17.788 | 66.8117 | 22.6215 | 93.6341 |
| 17.886 | 3.691 | 22.7462 | 4.06753 | 17.886 | 66.0172 | 22.7462 | 92.5206 |
| 18.01 | 3.613 | 22.9038 | 3.98157 | 18.01 | 65.0701 | 22.9038 | 91.1933 |
| 18.101 | 3.527 | 23.0196 | 3.8868 | 18.101 | 63.8422 | 23.0196 | 89.4725 |
| 18.189 | 3.452 | 23.1315 | 3.80415 | 18.189 | 62.7884 | 23.1315 | 87.9956 |
| 18.297 | 3.38 | 23.2688 | 3.7248 | 18.297 | 61.8439 | 23.2688 | 86.6718 |
| 18.372 | 3.318 | 23.3642 | 3.65648 | 18.372 | 60.9583 | 23.3642 | 85.4307 |
| 18.448 | 3.247 | 23.4609 | 3.57824 | 18.448 | 59.9007 | 23.4609 | 83.9485 |
| 18.541 | 3.174 | 23.5791 | 3.49779 | 18.541 | 58.8491 | 23.5791 | 82.4748 |
| 18.601 | 3.099 | 23.6554 | 3.41514 | 18.601 | 57.6445 | 23.6554 | 80.7866 |
| 18.689 | 3.028 | 23.7793 | 3.35696 | 18.689 | 56.5903 | 23.7793 | 79.8263 |
| 19.357 | 2.276 | 24.6293 | 2.52326 | 19.357 | 44.0565 | 24.6293 | 62.1462 |
| 19.802 | 1.648 | 25.1955 | 1.82704 | 19.802 | 32.6337 | 25.1955 | 46.0331 |
| 20.138 | 1.139 | 25.623 | 1.26274 | 20.138 | 22.9372 | 25.623 | 32.3552 |
| 20.358 | 0.764 | 25.9029 | 0.847001 | 20.358 | 15.5535 | 25.9029 | 21.9398 |
| 20.53 | 0.482 | 26.1218 | 0.534364 | 20.53 | 9.89546 | 26.1218 | 13.9585 |
| 20.628 | 0.294 | 26.2465 | 0.32594 | 20.628 | 6.06463 | 26.2465 | 8.55478 |
| 20.779 | 0 | 26.4386 | 0 | 20.779 | 0 | 26.4386 | 0 |

**Table 2**

| Voltage | Current | Voltage | Current | Voltage | Power | Voltage | Power |
| --- | --- | --- | --- | --- | --- | --- | --- |
| V | A | V | A | V | W | V | W |
| 20_MEAS | 20_MEAS | 20_STC | 20_STC | 20_MEAS_PWR | 20_MEAS_PWR | 20_STC_PWR | 20_STC_PWR |
| 0 | 3.029 | 0 | 2.98397 | 0 | 0 | 0 | 0 |
| 3.331 | 3.027 | 4.50488 | 2.982 | 3.331 | 10.0829 | 4.50488 | 13.4335 |
| 3.946 | 3.025 | 5.33661 | 2.98003 | 3.946 | 11.9367 | 5.33661 | 15.9032 |
| 6.17 | 3.001 | 8.34437 | 2.95639 | 6.17 | 18.5162 | 8.34437 | 24.6692 |
| 9.295 | 2.958 | 12.5706 | 2.91402 | 9.295 | 27.4946 | 12.5706 | 36.6312 |
| 12.274 | 2.87 | 16.5995 | 2.82733 | 12.274 | 35.2264 | 16.5995 | 46.9322 |
| 13.494 | 2.804 | 18.2494 | 2.76231 | 13.494 | 37.8372 | 18.2494 | 50.4106 |
| 14.013 | 2.747 | 18.9472 | 2.69896 | 14.013 | 38.4937 | 18.9472 | 51.1376 |
| 14.42 | 2.681 | 19.4975 | 2.63411 | 14.42 | 38.66 | 19.4975 | 51.3586 |
| 14.768 | 2.615 | 19.968 | 2.56927 | 14.768 | 38.6183 | 19.968 | 51.3032 |
| 15.052 | 2.567 | 20.352 | 2.52211 | 15.052 | 38.6385 | 20.352 | 51.3299 |
| 15.291 | 2.497 | 20.6752 | 2.45333 | 15.291 | 38.1816 | 20.6752 | 50.723 |
| 15.502 | 2.448 | 20.9605 | 2.40519 | 15.502 | 37.9489 | 20.9605 | 50.4139 |
| 15.713 | 2.381 | 21.2458 | 2.33936 | 15.713 | 37.4127 | 21.2458 | 49.7015 |
| 15.883 | 2.327 | 21.4753 | 2.28582 | 15.883 | 36.9597 | 21.4753 | 49.0885 |
| 16.007 | 2.273 | 21.6429 | 2.23277 | 16.007 | 36.3839 | 21.6429 | 48.3237 |
| 16.165 | 2.206 | 21.8565 | 2.16696 | 16.165 | 35.66 | 21.8565 | 47.3622 |
| 16.286 | 2.152 | 22.0202 | 2.11391 | 16.286 | 35.0475 | 22.0202 | 46.5487 |
| 16.38 | 2.104 | 22.1472 | 2.06676 | 16.38 | 34.4635 | 22.1472 | 45.7731 |
| 16.504 | 2.052 | 22.3149 | 2.01568 | 16.504 | 33.8662 | 22.3149 | 44.9798 |
| 16.574 | 1.998 | 22.4096 | 1.96264 | 16.574 | 33.1149 | 22.4096 | 43.9819 |
| 16.664 | 1.939 | 22.5328 | 1.90631 | 16.664 | 32.3115 | 22.5328 | 42.9544 |
| 16.764 | 1.888 | 22.668 | 1.85617 | 16.764 | 31.6504 | 22.668 | 42.0756 |
| 17.547 | 1.344 | 23.7268 | 1.32134 | 17.547 | 23.5832 | 23.7268 | 31.3511 |
| 18.008 | 0.918 | 24.3501 | 0.902522 | 18.008 | 16.5313 | 24.3501 | 21.9765 |
| 18.337 | 0.599 | 24.795 | 0.5889 | 18.337 | 10.9839 | 24.795 | 14.6018 |
| 18.541 | 0.372 | 25.0709 | 0.365728 | 18.541 | 6.89725 | 25.0709 | 9.16911 |
| 18.85 | 0 | 25.4887 | 0 | 18.85 | 0 | 25.4887 | 0 |

**Table 3**

| Voltage | Current | Voltage | Current | Voltage | Power | Voltage | Power |
| --- | --- | --- | --- | --- | --- | --- | --- |
| V | A | V | A | V | W | V | W |
| 22_MEAS | 22_MEAS | 22_STC | 22_STC | 22_MEAS_PWR | 22_MEAS_PWR | 22_STC_PWR | 22_STC_PWR |
| 0 | 3.444 | 0 | 3.54268 | 0 | 0 | 0 | 0 |
| 3.287 | 3.448 | 3.89473 | 3.54679 | 3.287 | 11.3336 | 3.89473 | 13.8138 |
| 3.45 | 3.446 | 4.08786 | 3.54474 | 3.45 | 11.8887 | 4.08786 | 14.4904 |
| 3.782 | 3.441 | 4.48124 | 3.53959 | 3.782 | 13.0139 | 4.48124 | 15.8618 |
| 5.411 | 3.433 | 6.41143 | 3.53137 | 5.411 | 18.576 | 6.41143 | 22.6411 |
| 8.974 | 3.407 | 10.6332 | 3.50462 | 8.974 | 30.5744 | 10.6332 | 37.2653 |
| 14.278 | 3.31 | 16.9178 | 3.40484 | 14.278 | 47.2602 | 16.9178 | 57.6025 |
| 15.865 | 3.236 | 18.7982 | 3.32872 | 15.865 | 51.3391 | 18.7982 | 62.5741 |
| 16.228 | 3.17 | 19.2284 | 3.26083 | 16.228 | 51.4428 | 19.2284 | 62.7004 |
| 16.472 | 3.114 | 19.5175 | 3.20322 | 16.472 | 51.2938 | 19.5175 | 62.5188 |
| 16.709 | 3.039 | 19.7983 | 3.12608 | 16.709 | 50.7787 | 19.7983 | 61.8909 |
| 16.876 | 2.975 | 19.9962 | 3.06024 | 16.876 | 50.2061 | 19.9962 | 61.1931 |
| 17.065 | 2.915 | 20.2201 | 2.99852 | 17.065 | 49.7445 | 20.2201 | 60.6305 |
| 17.187 | 2.839 | 20.3647 | 2.92035 | 17.187 | 48.7939 | 20.3647 | 59.4718 |
| 17.314 | 2.774 | 20.5151 | 2.85348 | 17.314 | 48.029 | 20.5151 | 58.5396 |
| 17.429 | 2.721 | 20.6503 | 2.79716 | 17.429 | 47.4243 | 20.6503 | 57.7622 |
| 17.527 | 2.661 | 20.7664 | 2.73548 | 17.527 | 46.6393 | 20.7664 | 56.8061 |
| 17.614 | 2.591 | 20.8695 | 2.66352 | 17.614 | 45.6379 | 20.8695 | 55.5863 |
| 17.718 | 2.522 | 20.9927 | 2.59259 | 17.718 | 44.6848 | 20.9927 | 54.4255 |
| 18.532 | 1.867 | 21.9572 | 1.91926 | 18.532 | 34.5992 | 21.9572 | 42.1414 |
| 19.02 | 1.32 | 22.5353 | 1.35695 | 19.02 | 25.1064 | 22.5353 | 30.5793 |
| 19.324 | 0.896 | 22.8955 | 0.921079 | 19.324 | 17.3143 | 22.8955 | 21.0886 |
| 19.545 | 0.584 | 23.1762 | 0.606204 | 19.545 | 11.4143 | 23.1762 | 14.0495 |
| 19.692 | 0.363 | 23.3505 | 0.376802 | 19.692 | 7.1482 | 23.3505 | 8.7985 |
| 19.764 | 0.215 | 23.4359 | 0.223175 | 19.764 | 4.24926 | 23.4359 | 5.23029 |
| 19.91 | 0 | 23.609 | 0 | 19.91 | 0 | 23.609 | 0 |

**Table 4**

| Voltage | Current | Voltage | Current | Voltage | Power | Voltage | Power |
| --- | --- | --- | --- | --- | --- | --- | --- |
| V | A | V | A | V | W | V | W |
| 25_MEAS | 25_MEAS | 25_STC | 25_STC | 25_MEAS_PWR | 25_MEAS_PWR | 25_STC_PWR | 25_STC_PWR |
| 0 | 1.263 | 0 | 1.23954 | 0 | 0 | 0 | 0 |
| 8.606 | 1.25 | 8.90926 | 1.22678 | 8.606 | 10.7575 | 8.90926 | 10.9297 |
| 52.239 | 1.173 | 54.0798 | 1.15121 | 52.239 | 61.2763 | 54.0798 | 62.2574 |
| 55.487 | 1.149 | 57.4423 | 1.12766 | 55.487 | 63.7546 | 57.4423 | 64.7753 |
| 58.387 | 1.119 | 60.4445 | 1.09822 | 58.387 | 65.3351 | 60.4445 | 66.3811 |
| 60.714 | 1.084 | 62.8535 | 1.06387 | 60.714 | 65.814 | 62.8535 | 66.8677 |
| 62.322 | 1.052 | 64.5181 | 1.03246 | 62.322 | 65.5627 | 64.5181 | 66.6124 |
| 63.789 | 1.018 | 65.9998 | 0.992211 | 63.789 | 64.9372 | 65.9998 | 65.4857 |
| 64.908 | 0.988 | 67.1576 | 0.962971 | 64.908 | 64.1291 | 67.1576 | 64.6708 |
| 65.884 | 0.955 | 68.1674 | 0.930807 | 65.884 | 62.9192 | 68.1674 | 63.4507 |
| 66.767 | 0.924 | 69.081 | 0.900593 | 66.767 | 61.6927 | 69.081 | 62.2138 |
| 67.589 | 0.894 | 69.9315 | 0.871353 | 67.589 | 60.4246 | 69.9315 | 60.935 |
| 68.996 | 0.836 | 71.3872 | 0.814822 | 68.996 | 57.6807 | 71.3872 | 58.1679 |
| 69.613 | 0.805 | 72.03 | 0.785199 | 69.613 | 56.0385 | 72.03 | 56.5579 |
| 70.181 | 0.778 | 72.6178 | 0.758863 | 70.181 | 54.6008 | 72.6178 | 55.1069 |
| 74.851 | 0.497 | 77.4499 | 0.484775 | 74.851 | 37.2009 | 77.4499 | 37.5458 |
| 78.832 | 0.177 | 81.5691 | 0.172646 | 78.832 | 13.9533 | 81.5691 | 14.0826 |
| 79.695 | 0.102 | 82.4621 | 0.099491 | 79.695 | 8.12889 | 82.4621 | 8.20424 |
| 80.679 | 0 | 83.4803 | 0 | 80.679 | 0 | 83.4803 | 0 |

**Table 5**

| Voltage | Current | Voltage | Current | Voltage | Power | Voltage | Power |
| --- | --- | --- | --- | --- | --- | --- | --- |
| V | A | V | A | V | W | V | W |
| 27_MEAS | 27_MEAS | 27_STC | 27_STC | 27_MEAS_PWR | 27_MEAS_PWR | 27_STC_PWR | 27_STC_PWR |
| 0 | 4.98 | 0 | 4.96353 | 0 | 0 | 0 | 0 |
| 3.771 | 4.972 | 4.75215 | 4.95555 | 3.771 | 18.7494 | 4.75215 | 23.5495 |
| 3.822 | 4.967 | 4.81642 | 4.95057 | 3.822 | 18.9839 | 4.81642 | 23.844 |
| 3.89 | 4.964 | 4.90211 | 4.94758 | 3.89 | 19.31 | 4.90211 | 24.2536 |
| 3.941 | 4.957 | 4.96638 | 4.9406 | 3.941 | 19.5355 | 4.96638 | 24.5369 |
| 4.056 | 4.956 | 5.1113 | 4.93961 | 4.056 | 20.1015 | 5.1113 | 25.2478 |
| 4.248 | 4.941 | 5.35325 | 4.92466 | 4.248 | 20.9894 | 5.35325 | 26.3629 |
| 5.182 | 4.922 | 6.53026 | 4.90572 | 5.182 | 25.5058 | 6.53026 | 32.0356 |
| 7.851 | 4.884 | 9.89541 | 4.87798 | 7.851 | 38.3443 | 9.89541 | 48.2696 |
| 10.765 | 4.824 | 13.5682 | 4.81806 | 10.765 | 51.9304 | 13.5682 | 65.3724 |
| 13.938 | 4.719 | 17.5675 | 4.71319 | 13.938 | 65.7734 | 17.5675 | 82.7988 |
| 15.104 | 4.646 | 19.0371 | 4.64028 | 15.104 | 70.1732 | 19.0371 | 88.3374 |
| 15.385 | 4.548 | 19.3913 | 4.5424 | 15.385 | 69.971 | 19.3913 | 88.0829 |
| 15.594 | 4.487 | 19.6547 | 4.48147 | 15.594 | 69.9703 | 19.6547 | 88.082 |
| 15.781 | 4.389 | 19.8904 | 4.38359 | 15.781 | 69.2628 | 19.8904 | 87.1914 |
| 15.945 | 4.319 | 20.0983 | 4.31683 | 15.945 | 68.8665 | 20.0983 | 86.7609 |
| 16.092 | 4.24 | 20.2836 | 4.23787 | 16.092 | 68.2301 | 20.2836 | 85.9592 |
| 16.254 | 4.146 | 20.4878 | 4.14391 | 16.254 | 67.3891 | 20.4878 | 84.8997 |
| 16.375 | 4.07 | 20.6403 | 4.06795 | 16.375 | 66.6463 | 20.6403 | 83.9638 |
| 16.498 | 4 | 20.7954 | 3.99799 | 16.498 | 65.992 | 20.7954 | 83.1396 |
| 16.598 | 3.919 | 20.9214 | 3.91703 | 16.598 | 65.0476 | 20.9214 | 81.9497 |
| 16.723 | 3.834 | 21.079 | 3.83207 | 16.723 | 64.116 | 21.079 | 80.7761 |
| 16.818 | 3.756 | 21.1987 | 3.75411 | 16.818 | 63.1684 | 21.1987 | 79.5823 |
| 17.743 | 2.902 | 22.3647 | 2.90054 | 17.743 | 51.4902 | 22.3647 | 64.8696 |
| 18.361 | 2.179 | 23.1436 | 2.1779 | 18.361 | 40.0086 | 23.1436 | 50.4046 |
| 18.805 | 1.573 | 23.7033 | 1.57221 | 18.805 | 29.5803 | 23.7033 | 37.2665 |
| 19.376 | 0.731 | 24.423 | 0.730632 | 19.376 | 14.1639 | 24.423 | 17.8442 |
| 19.527 | 0.463 | 24.6133 | 0.462767 | 19.527 | 9.041 | 24.6133 | 11.3902 |
| 19.629 | 0.282 | 24.7477 | 0.282653 | 19.629 | 5.53538 | 24.7477 | 6.99503 |
| 19.819 | 0 | 24.9873 | 0 | 19.819 | 0 | 24.9873 | 0 |

**Table 6**

| Voltage | Current | Voltage | Current | Voltage | Power | Voltage | Power |
| --- | --- | --- | --- | --- | --- | --- | --- |
| V | A | V | A | V | W | V | W |
| 28_MEAS | 28_MEAS | 28_STC | 28_STC | 28_MEAS_PWR | 28_MEAS_PWR | 28_STC_PWR | 28_STC_PWR |
| 0 | 2.485 | 0 | 2.37268 | 0 | 0 | 0 | 0 |
| 2.956 | 2.478 | 3.88919 | 2.366 | 2.956 | 7.32497 | 3.88919 | 9.20182 |
| 2.977 | 2.467 | 3.91682 | 2.3555 | 2.977 | 7.34426 | 3.91682 | 9.22605 |
| 3.056 | 2.443 | 4.02076 | 2.33258 | 3.056 | 7.46581 | 4.02076 | 9.37874 |
| 3.069 | 2.372 | 4.04254 | 2.29733 | 3.069 | 7.27967 | 4.04254 | 9.28705 |
| 3.116 | 2.311 | 4.10445 | 2.23825 | 3.116 | 7.20108 | 4.10445 | 9.18679 |
| 3.15 | 2.231 | 4.14924 | 2.16077 | 3.15 | 7.02765 | 4.14924 | 8.96554 |
| 16.017 | 1.995 | 21.2609 | 2.12293 | 16.017 | 31.9539 | 21.2609 | 45.1355 |
| 16.438 | 1.948 | 21.8198 | 2.07292 | 16.438 | 32.0212 | 21.8198 | 45.2306 |
| 16.682 | 1.886 | 22.1437 | 2.00694 | 16.682 | 31.4623 | 22.1437 | 44.441 |
| 16.823 | 1.841 | 22.3308 | 1.95906 | 16.823 | 30.9711 | 22.3308 | 43.7473 |
| 16.949 | 1.799 | 22.4981 | 1.91436 | 16.949 | 30.4913 | 22.4981 | 43.0695 |
| 17.08 | 1.744 | 22.672 | 1.85584 | 17.08 | 29.7875 | 22.672 | 42.0754 |
| 17.225 | 1.691 | 22.8644 | 1.79944 | 17.225 | 29.1275 | 22.8644 | 41.1431 |
| 17.265 | 1.645 | 22.7267 | 1.58027 | 17.265 | 28.4009 | 22.7267 | 35.9143 |
| 17.37 | 1.607 | 22.8649 | 1.54376 | 17.37 | 27.9136 | 22.8649 | 35.298 |
| 17.443 | 1.564 | 22.961 | 1.50246 | 17.443 | 27.2809 | 22.961 | 34.4979 |
| 17.519 | 1.517 | 23.0611 | 1.4573 | 17.519 | 26.5763 | 23.0611 | 33.607 |
| 17.592 | 1.464 | 23.1572 | 1.40639 | 17.592 | 25.7547 | 23.1572 | 32.568 |
| 17.646 | 1.422 | 23.2282 | 1.36604 | 17.646 | 25.0926 | 23.2282 | 31.7308 |
| 17.72 | 1.381 | 23.3256 | 1.32666 | 17.72 | 24.4713 | 23.3256 | 30.9451 |
| 17.778 | 1.341 | 23.402 | 1.28823 | 17.778 | 23.8403 | 23.402 | 30.1472 |
| 17.848 | 1.306 | 23.5177 | 1.27015 | 17.848 | 23.3095 | 23.5177 | 29.8711 |
| 17.897 | 1.265 | 23.5822 | 1.23028 | 17.897 | 22.6397 | 23.5822 | 29.0127 |
| 18.461 | 0.858 | 24.3254 | 0.83445 | 18.461 | 15.8395 | 24.3254 | 20.2983 |
| 18.811 | 0.554 | 24.7866 | 0.538794 | 18.811 | 10.4213 | 24.7866 | 13.3549 |
| 19.199 | 0.201 | 25.2978 | 0.195483 | 19.199 | 3.859 | 25.2978 | 4.9453 |
| 19.379 | 0 | 25.535 | 0 | 19.379 | 0 | 25.535 | 0 |

**Table 7**

| Voltage | Current | Voltage | Current | Voltage | Power | Voltage | Power |
| --- | --- | --- | --- | --- | --- | --- | --- |
| V | A | V | A | V | W | V | W |
| 30_MEAS | 30_MEAS | 30_STC | 30_STC | 30_MEAS_PWR | 30_MEAS_PWR | 30_STC_PWR | 30_STC_PWR |
| 0 | 2.641 | 0 | 2.77928 | 0 | 0 | 0 | 0 |
| 3.28 | 2.644 | 4.98526 | 2.78244 | 3.28 | 8.67232 | 4.98526 | 13.8712 |
| 6.897 | 2.588 | 10.4827 | 2.7235 | 6.897 | 17.8494 | 10.4827 | 28.5497 |
| 10.143 | 2.538 | 15.4163 | 2.67089 | 10.143 | 25.7429 | 15.4163 | 41.1752 |
| 13.008 | 2.452 | 19.7708 | 2.58038 | 13.008 | 31.8956 | 19.7708 | 51.0162 |
| 13.873 | 2.39 | 21.0855 | 2.51514 | 13.873 | 33.1565 | 21.0855 | 53.0329 |
| 14.273 | 2.332 | 21.7204 | 2.49185 | 14.273 | 33.2846 | 21.7204 | 54.1241 |
| 14.572 | 2.284 | 22.1754 | 2.44056 | 14.572 | 33.2824 | 22.1754 | 54.1205 |
| 14.856 | 2.223 | 22.6076 | 2.37538 | 14.856 | 33.0249 | 22.6076 | 53.7017 |
| 15.094 | 2.178 | 22.9698 | 2.3273 | 15.094 | 32.8747 | 22.9698 | 53.4576 |
| 15.303 | 2.114 | 23.2879 | 2.25891 | 15.303 | 32.3505 | 23.2879 | 52.6052 |
| 15.508 | 2.067 | 23.5998 | 2.20869 | 15.508 | 32.055 | 23.5998 | 52.1247 |
| 15.705 | 2.005 | 23.8996 | 2.14244 | 15.705 | 31.4885 | 23.8996 | 51.2035 |
| 15.878 | 1.963 | 24.1782 | 2.11393 | 15.878 | 31.1685 | 24.1782 | 51.1109 |
| 16.034 | 1.913 | 24.4158 | 2.06008 | 16.034 | 30.673 | 24.4158 | 50.2984 |
| 16.169 | 1.854 | 24.6213 | 1.99654 | 16.169 | 29.9773 | 24.6213 | 49.1576 |
| 16.429 | 1.762 | 25.0172 | 1.89747 | 16.429 | 28.9479 | 25.0172 | 47.4695 |
| 16.583 | 1.711 | 25.2517 | 1.84255 | 16.583 | 28.3735 | 25.2517 | 46.5276 |
| 17.554 | 1.196 | 26.7303 | 1.28795 | 17.554 | 20.9946 | 26.7303 | 34.4275 |
| 18.544 | 0.517 | 28.2219 | 0.5529 | 18.544 | 9.58725 | 28.2219 | 15.6039 |
| 18.798 | 0.319 | 28.6085 | 0.341151 | 18.798 | 5.99656 | 28.6085 | 9.75981 |
| 18.958 | 0.189 | 28.852 | 0.202124 | 18.958 | 3.58306 | 28.852 | 5.83167 |
| 19.18 | 0 | 29.1898 | 0 | 19.18 | 0 | 29.1898 | 0 |

**Table 8**

| Voltage | Current | Voltage | Current | Voltage | Power | Voltage | Power |
| --- | --- | --- | --- | --- | --- | --- | --- |
| V | A | V | A | V | W | V | W |
| 32_MEAS | 32_MEAS | 32_STC | 32_STC | 32_MEAS_PWR | 32_MEAS_PWR | 32_STC_PWR | 32_STC_PWR |
| 0 | 2.958 | 0 | 2.74757 | 0 | 0 | 0 | 0 |
| 3.104 | 2.962 | 4.3572 | 2.75129 | 3.104 | 9.19405 | 4.3572 | 11.9879 |
| 3.219 | 2.959 | 4.519 | 2.75131 | 3.219 | 9.52502 | 4.519 | 12.4332 |
| 3.362 | 2.96 | 4.71975 | 2.75224 | 3.362 | 9.95152 | 4.71975 | 12.9899 |
| 3.761 | 2.959 | 5.27989 | 2.75131 | 3.761 | 11.1288 | 5.27989 | 14.5266 |
| 8.473 | 2.899 | 11.8948 | 2.69553 | 8.473 | 24.5632 | 11.8948 | 32.0629 |
| 10.71 | 2.818 | 15.0353 | 2.62021 | 10.71 | 30.1808 | 15.0353 | 39.3956 |
| 11.913 | 2.743 | 16.7241 | 2.55048 | 11.913 | 32.6774 | 16.7241 | 42.6544 |
| 12.699 | 2.691 | 17.8275 | 2.50212 | 12.699 | 34.173 | 17.8275 | 44.6067 |
| 13.405 | 2.627 | 18.8186 | 2.44262 | 13.405 | 35.2149 | 18.8186 | 45.9667 |
| 13.877 | 2.567 | 19.478 | 2.38195 | 13.877 | 35.6223 | 19.478 | 46.3958 |
| 14.247 | 2.509 | 19.9974 | 2.32813 | 14.247 | 35.7457 | 19.9974 | 46.5566 |
| 14.523 | 2.455 | 20.3848 | 2.27803 | 14.523 | 35.654 | 20.3848 | 46.4371 |
| 14.813 | 2.389 | 20.7918 | 2.21678 | 14.813 | 35.3883 | 20.7918 | 46.091 |
| 15.077 | 2.339 | 21.1624 | 2.17039 | 15.077 | 35.2651 | 21.1624 | 45.9306 |
| 15.292 | 2.272 | 21.4642 | 2.10822 | 15.292 | 34.7434 | 21.4642 | 45.2511 |
| 15.663 | 2.167 | 21.9867 | 2.01284 | 15.663 | 33.9417 | 21.9867 | 44.2558 |
| 15.832 | 2.108 | 22.224 | 1.95804 | 15.832 | 33.3739 | 22.224 | 43.5154 |
| 15.983 | 2.062 | 22.4359 | 1.91531 | 15.983 | 32.9569 | 22.4359 | 42.9718 |
| 16.107 | 2.007 | 22.61 | 1.86423 | 16.107 | 32.3267 | 22.61 | 42.1501 |
| 16.248 | 1.946 | 22.8079 | 1.80756 | 16.248 | 31.6186 | 22.8079 | 41.2268 |
| 16.365 | 1.905 | 22.9721 | 1.76948 | 16.365 | 31.1753 | 22.9721 | 40.6488 |
| 16.473 | 1.856 | 23.1238 | 1.72397 | 16.473 | 30.5739 | 23.1238 | 39.8646 |
| 16.583 | 1.801 | 23.2782 | 1.67288 | 16.583 | 29.866 | 23.2782 | 38.9416 |
| 16.687 | 1.745 | 23.4242 | 1.62086 | 16.687 | 29.1188 | 23.4242 | 37.9674 |
| 17.521 | 1.234 | 24.5949 | 1.14622 | 17.521 | 21.6209 | 24.5949 | 28.191 |
| 18.001 | 0.832 | 25.2687 | 0.772813 | 18.001 | 14.9768 | 25.2687 | 19.528 |
| 18.335 | 0.537 | 25.7375 | 0.498799 | 18.335 | 9.8459 | 25.7375 | 12.8378 |
| 18.547 | 0.329 | 26.0351 | 0.305596 | 18.547 | 6.10196 | 26.0351 | 7.95621 |
| 18.676 | 0.196 | 26.2162 | 0.182057 | 18.676 | 3.6605 | 26.2162 | 4.77284 |
| 18.839 | 0 | 26.445 | 0 | 18.839 | 0 | 26.445 | 0 |

**Table 9**

| Voltage | Current | Voltage | Current | Voltage | Power | Voltage | Power |
| --- | --- | --- | --- | --- | --- | --- | --- |
| V | A | V | A | V | W | V | W |
| 36_MEAS | 36_MEAS | 36_STC | 36_STC | 36_MEAS_PWR | 36_MEAS_PWR | 36_STC_PWR | 36_STC_PWR |
| 0 | 5.126 | 0 | 5.22895 | 0 | 0 | 0 | 0 |
| 3.901 | 5.122 | 4.82318 | 5.22819 | 3.901 | 19.9809 | 4.82318 | 25.2165 |
| 3.964 | 5.119 | 4.90107 | 5.22513 | 3.964 | 20.2917 | 4.90107 | 25.6087 |
| 4.266 | 5.116 | 5.27446 | 5.22206 | 4.266 | 21.8249 | 5.27446 | 27.5436 |
| 5.182 | 5.105 | 6.407 | 5.21084 | 5.182 | 26.4541 | 6.407 | 33.3858 |
| 7.94 | 5.075 | 9.81698 | 5.18021 | 7.94 | 40.2955 | 9.81698 | 50.854 |
| 11.275 | 5.01 | 13.9404 | 5.11387 | 11.275 | 56.4877 | 13.9404 | 71.2891 |
| 14.309 | 4.892 | 17.6925 | 4.99659 | 14.309 | 69.9996 | 17.6925 | 88.4024 |
| 15.048 | 4.827 | 18.6063 | 4.9302 | 15.048 | 72.6367 | 18.6063 | 91.7327 |
| 15.345 | 4.726 | 18.9735 | 4.82705 | 15.345 | 72.5205 | 18.9735 | 91.5859 |
| 15.552 | 4.66 | 19.2294 | 4.75963 | 15.552 | 72.4723 | 19.2294 | 91.5251 |
| 15.772 | 4.566 | 19.5015 | 4.66362 | 15.772 | 72.015 | 19.5015 | 90.9475 |
| 15.951 | 4.498 | 19.7228 | 4.59417 | 15.951 | 71.7476 | 19.7228 | 90.6099 |
| 16.133 | 4.395 | 19.9478 | 4.48897 | 16.133 | 70.9045 | 19.9478 | 89.5452 |
| 16.267 | 4.317 | 20.1114 | 4.4037 | 16.267 | 70.2246 | 20.1114 | 88.5645 |
| 16.382 | 4.25 | 20.2536 | 4.33535 | 16.382 | 69.6235 | 20.2536 | 87.8063 |
| 16.538 | 4.167 | 20.4464 | 4.25069 | 16.538 | 68.9138 | 20.4464 | 86.9113 |
| 16.749 | 3.992 | 20.7073 | 4.07217 | 16.749 | 66.862 | 20.7073 | 84.3236 |
| 16.865 | 3.934 | 20.8507 | 4.01301 | 16.865 | 66.3469 | 20.8507 | 83.674 |
| 17.823 | 3.053 | 22.0351 | 3.11431 | 17.823 | 54.4136 | 22.0351 | 68.6242 |
| 18.453 | 2.294 | 22.8178 | 2.34479 | 18.453 | 42.3312 | 22.8178 | 53.503 |
| 18.906 | 1.665 | 23.378 | 1.70186 | 18.906 | 31.4785 | 23.378 | 39.7861 |
| 19.224 | 1.161 | 23.7712 | 1.1867 | 19.224 | 22.3191 | 23.7712 | 28.2094 |
| 19.487 | 0.779 | 24.0964 | 0.796246 | 19.487 | 15.1804 | 24.0964 | 19.1867 |
| 19.673 | 0.495 | 24.3264 | 0.505959 | 19.673 | 9.73813 | 24.3264 | 12.3082 |
| 19.783 | 0.306 | 24.4624 | 0.312775 | 19.783 | 6.0536 | 24.4624 | 7.65123 |
| 19.95 | 0 | 24.6689 | 0 | 19.95 | 0 | 24.6689 | 0 |

**Table 10**

| Voltage | Current | Voltage | Current | Voltage | Power | Voltage | Power |
| --- | --- | --- | --- | --- | --- | --- | --- |
| V | A | V | A | V | W | V | W |
| 39_MEAS | 39_MEAS | 39_STC | 39_STC | 39_MEAS_PWR | 39_MEAS_PWR | 39_STC_PWR | 39_STC_PWR |
| 0 | 5.968 | 0 | 6.97767 | 0 | 0 | 0 | 0 |
| 4.177 | 5.959 | 5.46428 | 6.96715 | 4.177 | 24.8907 | 5.46428 | 38.0705 |
| 4.305 | 5.959 | 5.63132 | 6.96127 | 4.305 | 25.6535 | 5.63132 | 39.2012 |
| 4.933 | 5.948 | 6.4528 | 6.94842 | 4.933 | 29.3415 | 6.4528 | 44.8368 |
| 8.317 | 5.814 | 10.8794 | 6.79188 | 8.317 | 48.355 | 10.8794 | 73.8914 |
| 9.385 | 5.714 | 12.2764 | 6.67506 | 9.385 | 53.6259 | 12.2764 | 81.9458 |
| 10.09 | 5.622 | 13.1986 | 6.56759 | 10.09 | 56.726 | 13.1986 | 86.6831 |
| 10.675 | 5.537 | 13.9639 | 6.46829 | 10.675 | 59.1075 | 13.9639 | 90.3223 |
| 11.214 | 5.451 | 14.6689 | 6.36783 | 11.214 | 61.1275 | 14.6689 | 93.4091 |
| 11.705 | 5.362 | 15.3084 | 6.25029 | 11.705 | 62.7622 | 15.3084 | 95.6817 |
| 12.159 | 5.268 | 15.9021 | 6.14072 | 12.159 | 64.0536 | 15.9021 | 97.6505 |
| 12.55 | 5.182 | 16.4135 | 6.04047 | 12.55 | 65.0341 | 16.4135 | 99.1452 |
| 12.889 | 5.091 | 16.8569 | 5.93439 | 12.889 | 65.6179 | 16.8569 | 100.035 |
| 13.201 | 5.012 | 17.2649 | 5.84231 | 13.201 | 66.1634 | 17.2649 | 100.867 |
| 13.487 | 4.915 | 17.639 | 5.72924 | 13.487 | 66.2886 | 17.639 | 101.058 |
| 13.716 | 4.839 | 17.9384 | 5.64065 | 13.716 | 66.3717 | 17.9384 | 101.184 |
| 13.94 | 4.743 | 18.2266 | 5.5115 | 13.94 | 66.1174 | 18.2266 | 100.456 |
| 14.151 | 4.669 | 18.5025 | 5.42551 | 14.151 | 66.071 | 18.5025 | 100.385 |
| 14.357 | 4.569 | 18.7718 | 5.3093 | 14.357 | 65.5971 | 18.7718 | 99.6652 |
| 14.521 | 4.501 | 18.9862 | 5.23029 | 14.521 | 65.359 | 18.9862 | 99.3034 |
| 14.698 | 4.406 | 19.2177 | 5.11989 | 14.698 | 64.7594 | 19.2177 | 98.3924 |
| 14.87 | 4.315 | 19.4425 | 5.01415 | 14.87 | 64.1641 | 19.4425 | 97.4878 |
| 14.989 | 4.252 | 19.5981 | 4.94094 | 14.989 | 63.7332 | 19.5981 | 96.8333 |
| 15.147 | 4.171 | 19.8047 | 4.84682 | 15.147 | 63.1781 | 19.8047 | 95.9899 |
| 15.285 | 4.072 | 19.9852 | 4.73178 | 15.285 | 62.2405 | 19.9852 | 94.5653 |
| 16.486 | 3.184 | 21.5555 | 3.6999 | 16.486 | 52.4914 | 21.5555 | 79.753 |
| 17.295 | 2.392 | 22.6132 | 2.77957 | 17.295 | 41.3696 | 22.6132 | 62.8551 |
| 17.86 | 1.75 | 23.352 | 2.03355 | 17.86 | 31.255 | 23.352 | 47.4874 |
| 18.293 | 1.223 | 23.9181 | 1.42116 | 18.293 | 22.3723 | 23.9181 | 33.9915 |
| 18.59 | 0.822 | 24.3065 | 0.955187 | 18.59 | 15.281 | 24.3065 | 23.2172 |
| 18.79 | 0.532 | 24.568 | 0.618199 | 18.79 | 9.99628 | 24.568 | 15.1879 |
| 18.937 | 0.329 | 24.7539 | 0.381164 | 18.937 | 6.23027 | 24.7539 | 9.43528 |
| 19.16 | 0 | 25.0454 | 0 | 19.16 | 0 | 25.0454 | 0 |

**Table 11**

| Voltage | Current | Voltage | Current | Voltage | Power | Voltage | Power |
| --- | --- | --- | --- | --- | --- | --- | --- |
| V | A | V | A | V | W | V | W |
| 40_MEAS | 40_MEAS | 40_STC | 40_STC | 40_MEAS_PWR | 40_MEAS_PWR | 40_STC_PWR | 40_STC_PWR |
| 0 | 4.814 | 0 | 5.50293 | 0 | 0 | 0 | 0 |
| 3.633 | 4.795 | 4.808 | 5.48121 | 3.633 | 17.4202 | 4.808 | 26.3536 |
| 3.727 | 4.792 | 4.9324 | 5.47778 | 3.727 | 17.8598 | 4.9324 | 27.0186 |
| 3.804 | 4.785 | 5.0343 | 5.46978 | 3.804 | 18.2021 | 5.0343 | 27.5365 |
| 3.945 | 4.771 | 5.2209 | 5.45377 | 3.945 | 18.8216 | 5.2209 | 28.4736 |
| 4.842 | 4.745 | 6.40801 | 5.42405 | 4.842 | 22.9753 | 6.40801 | 34.7574 |
| 8.604 | 4.623 | 11.3867 | 5.28459 | 8.604 | 39.7763 | 11.3867 | 60.1743 |
| 10.965 | 4.554 | 14.5151 | 5.22192 | 10.965 | 49.9346 | 14.5151 | 75.7969 |
| 13.466 | 4.459 | 17.8259 | 5.11299 | 13.466 | 60.0449 | 17.8259 | 91.1435 |
| 14.383 | 4.379 | 19.0398 | 5.02125 | 14.383 | 62.9832 | 19.0398 | 95.6036 |
| 14.872 | 4.306 | 19.6871 | 4.93755 | 14.872 | 64.0388 | 19.6871 | 97.206 |
| 15.254 | 4.229 | 20.1928 | 4.84925 | 15.254 | 64.5092 | 20.1928 | 97.92 |
| 15.646 | 4.149 | 20.7117 | 4.75752 | 15.646 | 64.9153 | 20.7117 | 98.5364 |
| 15.87 | 4.07 | 21.0082 | 4.66693 | 15.87 | 64.5909 | 21.0082 | 98.044 |
| 16.045 | 3.998 | 21.2468 | 4.602 | 16.045 | 64.1479 | 21.2468 | 97.7776 |
| 16.22 | 3.909 | 21.4785 | 4.49955 | 16.22 | 63.404 | 21.4785 | 96.6437 |
| 16.341 | 3.835 | 21.6387 | 4.41437 | 16.341 | 62.6677 | 21.6387 | 95.5214 |
| 16.469 | 3.769 | 21.8082 | 4.3384 | 16.469 | 62.0717 | 21.8082 | 94.6129 |
| 16.603 | 3.681 | 21.9857 | 4.23711 | 16.603 | 61.1156 | 21.9857 | 93.1556 |
| 16.83 | 3.529 | 22.2863 | 4.06214 | 16.83 | 59.3931 | 22.2863 | 90.53 |
| 16.915 | 3.467 | 22.3988 | 3.99078 | 16.915 | 58.6443 | 22.3988 | 89.3887 |
| 17 | 3.393 | 22.5114 | 3.9056 | 17 | 57.681 | 22.5114 | 87.9204 |
| 17.187 | 3.24 | 22.759 | 3.72948 | 17.187 | 55.6859 | 22.759 | 84.8793 |
| 17.292 | 3.169 | 22.898 | 3.64776 | 17.292 | 54.7983 | 22.898 | 83.5265 |
| 17.375 | 3.11 | 23.008 | 3.57984 | 17.375 | 54.0362 | 23.008 | 82.3649 |
| 18.127 | 2.331 | 24.0037 | 2.68316 | 18.127 | 42.254 | 24.0037 | 64.4058 |
| 18.646 | 1.692 | 24.691 | 1.94762 | 18.646 | 31.549 | 24.691 | 48.0887 |
| 19.011 | 1.188 | 25.1789 | 1.37044 | 19.011 | 22.5851 | 25.1789 | 34.5062 |
| 19.291 | 0.799 | 25.5498 | 0.921702 | 19.291 | 15.4135 | 25.5498 | 23.5493 |
| 19.481 | 0.514 | 25.8014 | 0.592935 | 19.481 | 10.0132 | 25.8014 | 15.2986 |
| 19.592 | 0.313 | 25.9484 | 0.361067 | 19.592 | 6.1323 | 25.9484 | 9.36913 |
| 19.779 | 0 | 26.1961 | 0 | 19.779 | 0 | 26.1961 | 0 |
