# Supplementary material for: Visual and electrical degradation data of five years aged rooftop photovoltaic modules
Source: Data Brief. 2020 May 24;31:105762. doi: 10.1016/j.dib.2020.105762 (PMC7267713; doi:10.1016/j.dib.2020.105762)
Supplement: Supplementary file 2 [file mmc2.docx]

**PV Modules Specifications**

**New PV Modules (Used as reference)**

| **Reference Panel 1** |  |
| --- | --- |
| Solar panel | 100W Mono Silicon |
| Number of cell and area | 15.5cm × 9.3cm × rectangular 36 cell =0.52m^2^ |
| Solar panel type | Mono crystalline |
| Year | New/2019 |
| Electrical Data | V_oc_=22V, I_sc_=5.88A, V_mp_=18V, I_mp_=5.54A |

**
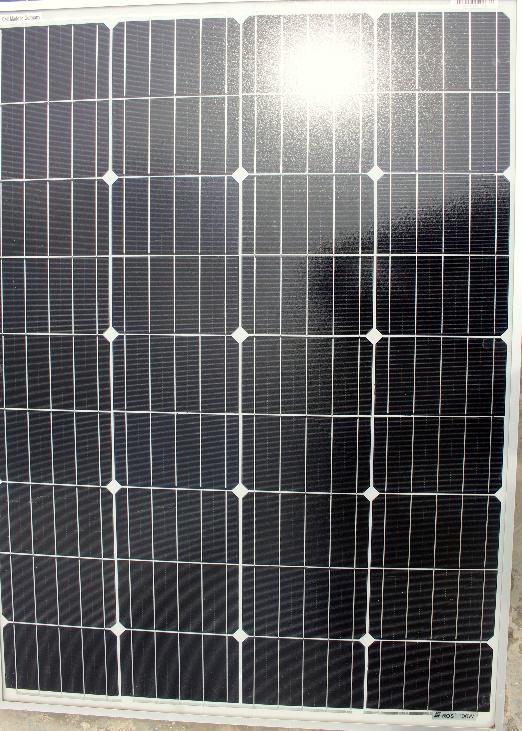
**

**PV 01 Mono Si 100W New Panel**

| **Reference Panel 2** |  |
| --- | --- |
| Solar panel | 100W Poly Silicon |
| Number of cell and area | 15.5cm × 6cm × 68 cell = 0.63m^2^ |
| Solar panel type | Poly crystalline |
| Year | New/2019 |
| Electrical Data | V_oc_=22.5V, I_sc_=5.76A, V_mp_=18.35V, I_mp_=5.44A |

**
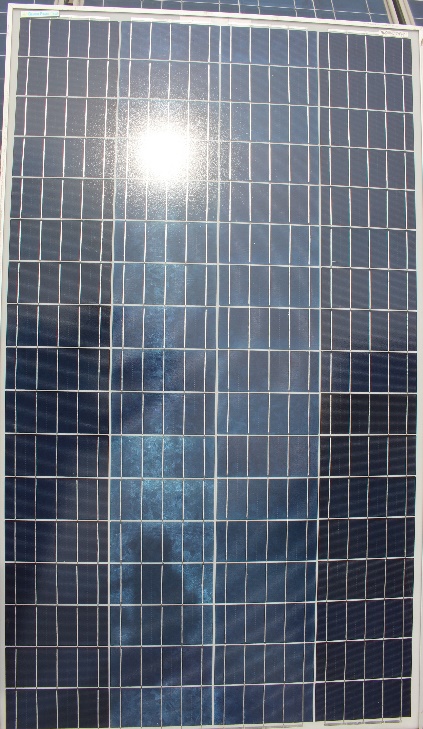
**

**PV 03 Poly Si 100W New Panel**

**Site 1**

| **Solar Power plant Site 1** |  |
| --- | --- |
| Installed Capacity | 5000W |
| Solar panel | 100 Panel each 50W |
| Number of cell and area | 15.4cm × 6.4cm × 36 cell = 0.35m^2^ |
| Solar panel type | Polycrystalline |
| Installation year | 2014 |
| Type of installation | On Grid |
| Electrical Data | V_oc_=21.6V, I_sc_=3.29A, V_mp_=17.3V, I_mp_=2.94A |


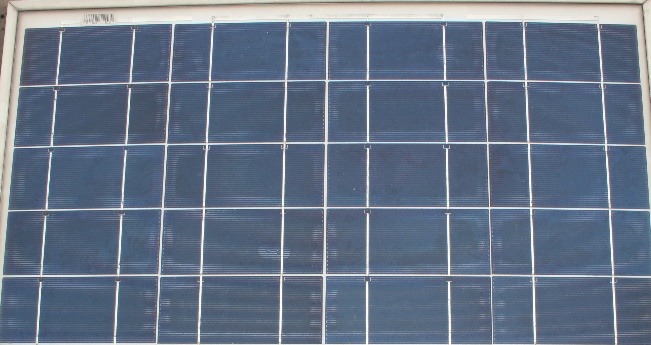


**PV 02 Poly Si 50W five year old panel**

**Site 2**

| **Solar Power plant Site 2** |  |
| --- | --- |
| Installed Capacity | 2550W |
| Solar panel | 30 Panel each 85W |
| Number of cell and area | 12.5cm × 12.5cm × 36 cell = 0.56m^2^ |
| Solar panel type | Mono crystalline |
| Installation year | 2014 |
| Type of installation | On Grid |
| Electrical Data | V_oc_=21.5V, I_sc_=5.42A, V_mp_=17.2V, I_mp_=4.94A |

**
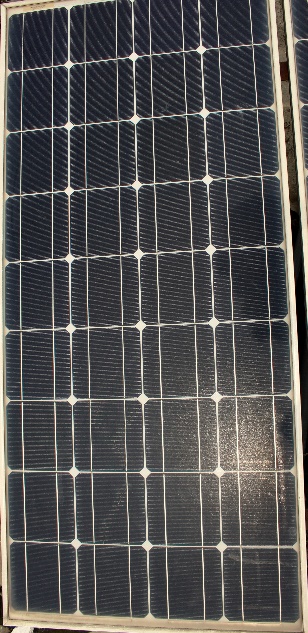
**

**PV 05 Mono Si 100W five year old panel**

**Site 3**

| **Solar Power plant Site 3** |  |
| --- | --- |
| Installed Capacity | 2550W |
| Solar panel | 17 Panel each 150W |
| Number of cell and area | 14cm × 14cm × 36 cell = 0.71m^2^ |
| Solar panel type | Polycrystalline |
| Installation year | 2013 |
| Type of installation | Off Grid |
| Electrical Data | V_oc_=21.6V, I_sc_=8.85A, V_mp_=17.2V, I_mp_=8.72A |


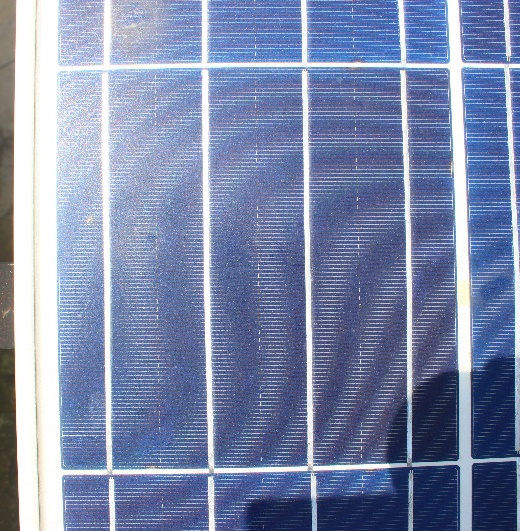


**PV 06 Poly Si 150W five year old panel**

**Site 4**

| **Solar Power plant Site 3** |  |
| --- | --- |
| Installed Capacity | 5000W |
| Solar panel | 50 Panels each 100W |
| Number of cell and area | 96.3cm × 93cm =0.90m^2^ single cell |
| Solar panel type | Amorphous Silicon thin film |
| Installation year | 2015 |
| Type of installation | Off Grid |
| Electrical Data | V_oc_=71.0V, I_sc_=2.25A, V_mp_=53.5V, I_mp_=1.87A |

**
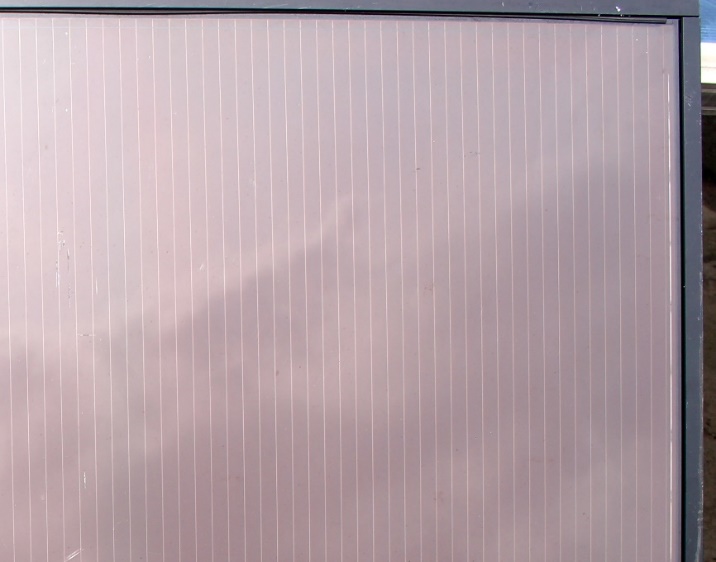
**

**PV 04 Amorphous Silicon thin film 100W five year old panel**
